# The role of cornulin (CRNN) in the progression of cutaneous squamous cell carcinoma involving AKT activation in SCL-1

**DOI:** 10.1371/journal.pone.0308243

**Published:** 2024-09-18

**Authors:** Changji Li, Peiwen Yang, Xin Wang, Hongbao Li, Huiling Jing, Yan Zheng, Yuzhu Hou

**Affiliations:** 1 Department of Pathogenic Microbiology and Immunology, School of Basic Medical Sciences, Xi’an Jiaotong University, Xi’an, Shaanxi, China; 2 Department of Dermatology, Shanghai General Hospital Jiuquan Hospital (Jiuquan City People’s Hospital), Jiuquan, Gansu, China; 3 Department of Dermatology, the First Affiliated Hospital, School of Medicine, Xi’an Jiaotong University, Xi’an, Shaanxi, China; 4 Department of Dermatology, the Second Affiliated Hospital, School of Medicine, Xi’an Jiaotong University, Xi’an, Shaanxi, China; 5 Department of Physiology and Pathophysiology, School of Basic Medical Sciences, Xi’an Jiaotong University Health Science Center, Xi’an, China; 6 Department of Dermatology, Xi’an Hospital of Traditional Chinese Medicine, Xi’an, China; Eskisehir Osmangazi University: Eskisehir Osmangazi Universitesi, TÜRKIYE

## Abstract

Cutaneous squamous cell carcinoma (cSCC) is the second most common non-melanoma skin cancer that has been on the rise in recent times, particularly among older individuals. Cornulin (CRNN) is increasingly recognized as an oncogene involved in developing various types of tumors. However, the precise contribution to cSCC remains unclear. Our study observed a significant increase in CRNN expression in cSCC samples compared to healthy skin. CRNN expression in the SCL-1 cell line derived from cSCC was reduced, leading to a halt in cell growth during the transition from the G1 phase to the S phase. This reduction inhibits cell division, promotes cell death, and decreases cell invasion and migration. CRNN overexpression has been found to enhance cell growth and prevent cells from undergoing natural cell death, and the cancer-promoting effects of CRNN are linked to AKT activation. Using a mouse xenograft model, we demonstrated that the inhibition of CRNN led to a decline in cSCC tumor growth in a living organism, providing evidence of CRNN’s involvement in cSCC occurrence and development. This study establishes a foundation for evaluating the effectiveness of CRNN in treating cSCC, enabling further investigation in this area.

## Introduction

Cutaneous squamous cell carcinoma (cSCC) is a slow-growing malignant tumor of keratinizing cells in the epidermis and appendages. They can become aggressive, spread to other body parts, and cause extensive damage. CSCC is the second most common type of skin cancer globally, after basal cell carcinoma [1, 2]. Studies indicate that individuals diagnosed with cSCC and lymph node metastasis have a 5-year survival rate ranging from 26% to 34% [3, 4]. Treating cSCCs is challenging due to the limited targeted therapy options and the high likelihood of recurrence and metastasis in primary cSCCs [5, 6]. Therefore, further investigation is warranted to improve existing approaches for the management and care of cSCC.

Cornulin (CRNN), also known as c1orf10 and SEP53, was initially identified as a new human gene in esophageal cells using differential display PCR [7, 8]. SEP53 earned its name from being called C1orf10 because of its robust synthesis following exposure to heat and its potential to produce a 53 kDa Ca2+-binding protein [9]. CRNN, the protein product of C1orf10, shares structural similarities with members of the epidermal differentiation complex (EDC) fusion gene family. EDC is critical for epidermal differentiation. Contzler et al. coined “cornulin” to describe this protein [10]. The CRNN is located in the EDC, encoding chromosome 1q21. Previous studies have displayed that CRNN is primarily localized in the cytoplasmic and perinuclear regions [10, 11]. CRNN over-expression has been detected in various squamous cell types, including oral, cervical, foreskin, skin, meibomian gland, thyroid follicular, and esophageal cells [10, 12–22]. Apart from eczema [14], earlier studies have demonstrated that CRNN is downregulated in various types of cancers, including esophageal squamous cell carcinoma, oral squamous cell carcinoma, cervical squamous cell carcinoma, head and neck squamous cell carcinoma, and thyroid cancer [11, 13, 15, 17, 19, 22–25]. However, their role in cSCC remains unclear. Using siRNA and LV-CRNN, we investigated the CRNN expression in samples from cSCC patients and null mice and explored the biological roles of CRNN. Our study observed a positive association between CRNN expression and cSCC. We found that CRNN contributed to the cSCC progression by suppressing the activation of the AKT signaling pathway.

## Materials and methods

### Reagents

Dimethyl sulfoxide (DMSO), 3-[4,5-dimethylthiazol-2-yl]-2,5-diphenyl-tetrazolium bromide (MTT), propidium iodide (PI), and RNase A were purchased from Sigma-Aldrich (St. Louis., MO, USA). CRNN antibody (11799-1-AP) was acquired from Protein tech (Chicago, IL, USA). Antibodies against Akt (pan) (#4691) and phospho-Akt (#2965) were acquired from Cell Signaling Technology (Danvers, MA, USA). Mouse monoclonal antibodies against cyclin D1 (sc-246) and β-Actin (sc-47778) were received from Santa Cruz Biotechnology (Santa Cruz, CA, USA). Dulbecco’s modified Eagle’s medium (DMEM) and fetal bovine serum (FBS) were provided by Invitrogen (Carlsbad, CA, USA). Polyvinylidene difluoride (PVDF) membranes for Western blotting analysis were obtained from Roche Diagnostics (Mannheim, Germany).

### Ethical issues

The utilization of archived tissue specimens and collection of normal skin and cSCC tissues was approved by the Institutional Ethics Committee of Xi’an Jiaotong University. The study adhered to the principles of the Declaration of Helsinki. All patients provided written informed consent for tissue procurement before the commencement of the study. The mouse experiments were approved by the Institutional Ethics Committee of Xi’an Jiaotong University and conducted according to institutional guidelines.

### Patient samples

From August 1, 2016 to July31, 2017, patient samples were obtained from the Department of Dermatology Tissue Bank at the Second Affiliated Hospital of Xi’an Jiaotong University. A total of 116 specimens were collected from individuals who had undergone cosmetic surgeries. These specimens included 57 healthy skin tissue samples(31 males and 26 females, aged 22–59 years) and 59 cSCC tissue samples (32 males and 27 females, aged 46–87 years;12 cases of poorly differentiated cSCC and 47 cases of well differentiated cSCC). Staining results were independently reviewed by two experienced pathologists. All participants in this study received ethical approval from the Institutional Ethics Committee of Xi’an Jiaotong University and provided written informed consent for tissue procurement prior to the commencement of the study.

### Western blotting

Total proteins were harvested from the cellular material using a lysis solution containing 50mM Tris-HCl (pH7.5), 15mM EGTA, 100mM NaCl, and 0.1% Triton X-100, supplemented with a protease inhibitor cocktail (Roche Diagnostics in Risch-Rotkreuz, Switzerland). The protein concentration was determined using the BCA Protein Assay Kit (Pierce, Rockford, IL). SDS-PAGE separated the proteins and subsequently transferred them to a PVDF membrane. The membrane was then incubated with a primary antibody targeting the specific protein of interest, followed by incubation with a secondary antibody conjugated with horseradish peroxidase. Protein detection was performed using an enhanced chemiluminescence system.

### Immunohistochemistry

Immunoperoxidase staining protocol [26] was used for immunohistochemical staining. A scoring system was employed to quantify the results of the staining evaluations, independently performed by two pathologists using a microscope. The assessment involved different levels, denoted as 0, 1, 2, 3, and 4, representing the proportion of cells exhibiting positive staining: less than 5%, 6%–25%, 26%–50%, 51%–75%, and greater than 75%, respectively. The scoring system for the staining intensity ranged from no color (0) to light yellow (1), yellow-brown (2), and dark brown (3). The aggregate score for each microscopic field was calculated by multiplying the aforementioned scores. The final CRNN expression score for each slide was determined by averaging the scores for the five fields. The total scores ranged from 0 to 12, indicating different levels of expression for CRNN, including negative (-), weak positive (+), moderate positive (++), and strong positive (+++).

### Transient transfection of CRNN siRNA

GenePharma (Shanghai, China) synthesized siRNA oligonucleotides using the sequences listed in Table 1. Lipofectamine 2000 Transfection Reagent (Invitrogen, Carlsbad, CA) was used to transfer the siRNA into the cells, following the manufacturer’s recommended protocol.

**Table 1:** The sequences of si-RNAs used in this study.

Name	Sequences
h-CRNN1	Forward: 5’-GGUUGGUGAGGAAUGGGUUTT-3’
	Reverse: 5’-AACCCAUUCCUCACCAACCTT-3’
h-CRNN2	Forward: 5’-GGAAUCAGACAACAGAGAUTT-3’
	Reverse: 5’-AUCUCUGUUGUCUGAUUCCTT-3’
Negative Control	Forward: 5’-UUCUUCGAACGUGUCACGUTT-3
	Reverse: 5’-ACGUGACACGUUCGGAGAATT-3’

### Cell culture and cell viability assay

The SCL-1 cells were cultured in Dulbecco’s modified Eagle’s medium (DMEM) supplemented with 10% heat-inactivated fetal bovine serum (FBS), penicillin (100 U/mL), and streptomycin (100 μg/mL) in a humidified atmosphere of 5% CO_2_ at 37°C. Cell viability was evaluated using the 3-(4,5-dimethylthiazol-2-yl)-2,5-diphenyltetrazolium bromide (MTT) assay (Sigma, USA) to determine the effect of siRNA on cell growth. Cells were seeded at a density of 5×104cells/well in a 96-well culture plate and transfected with siRNA the following day. After 24 h of incubation, 20 μLMTT solution (5 mg/mL) was added to each well. Following a 4 h incubation at 37°C, the supernatant was replaced with 150 μL of DMSO, and the absorbance at 490 nm was quantified for each well. The percentage of SCL-1 cell survival was determined by comparing the absorbance of wells treated with siRNA to those without siRNA. Each data point was obtained from five experiments. At each time point, the average values from three independent tests were used for plotting.

### Lentivirus transduction

The lentiviruses employed for transduction, including the control non-targeting VEC and targeting CRNN lentiviruses, were procured from GenePharma (Shanghai, China). To perform the transduction process, 10 μL of the virus suspension with a titer of 1×109 transduction units/mL was added to 1 mL of complete medium. After a 48-h transduction period, the cells were subjected to selection using a medium containing 5 mg/mL puromycin for two weeks.

### Cell cycle analysis

Cellular DNA content was evaluated by propidium iodide (PI) staining after treating the cells with siRNA for 48 h, which involved sequential steps, including trypsin hydrolysis, two washes with phosphate-buffered saline (PBS) at a low temperature, and fixation with immersion in 70% ethanol at 4°C. The cells were then granulated twice, followed by two rinses with1% FBS/PBS solution. The sample was incubated with 1 mg/mL RNase A at 37°C for 30 min to eliminate double-stranded RNA. The sample was stained with 1 μM DAPI for 10 min at room temperature to visualize DNA content. Flow cytometric analysis of DNA content was conducted using a FACSCalibur flow cytometer (BD Biosciences, USA). ModFit software was employed to examine the distribution of the G0-G1 phase in the cell cycle S. Three separate trials were conducted to examine the G2-M phases.

### Animal studies

On November 23, 2016, female BALB/c/ nude mice (4–5 weeks old) were obtained from Hunan Slack King Experimental Animal Co., Ltd. (Hunan Province, China) under the license number SCXK (Xiang)2013-0004. The room temperature for nude mice is usually 26–28°C, and the relative humidity should be maintained at 40%-60%, with sufficient ventilation to keep the air fresh and clean. A 12-h alternating light and dark cycle was provided, special cages were used, and balanced nutrition, food and water were provided. During the nude-mouse-rearing process, strict hygiene and operational norms should be followed. Five mice were injected with LV-CRNN SCL-1cells beneath the skin of their left legs, and LV-VEC SCL-1 cells beneath the skin of their right legs, respectively. Every72 h, the tumor dimensions were assessed using a modern digital micro caliper. Tumor size was calculated using the formula: tumor volume = length× width × width/2. After 15 days, the mice were humanely euthanized by CO2 inhalation following the research experiment. During the procedure, the tumors were surgically removed, and their weights were measured. None of the mice died before meeting the criteria for euthanasia. All animal experiments, including Western blotting and histological analyses, were conducted following protocols approved by the Institutional Animal Care and Use Committee of Xi’an Jiaotong University.

### Statistical analysis

All data are reported as mean ± SEM. Statistical analysis was performed using the SPSS software package (SPSS, Chicago, IL, USA). Immunohistochemical analysis was performed using Pearson’s chi-square test and Wilcoxon tests. Student’s t-test was used to compare two groups, while one-way analysis of variance (ANOVA) was used for experiments involving more than two groups. A statistically significant difference was considered statistically less than 0.05.

## Results

### Upregulation of CRNN protein expression in cSCC

**Fig 1:**
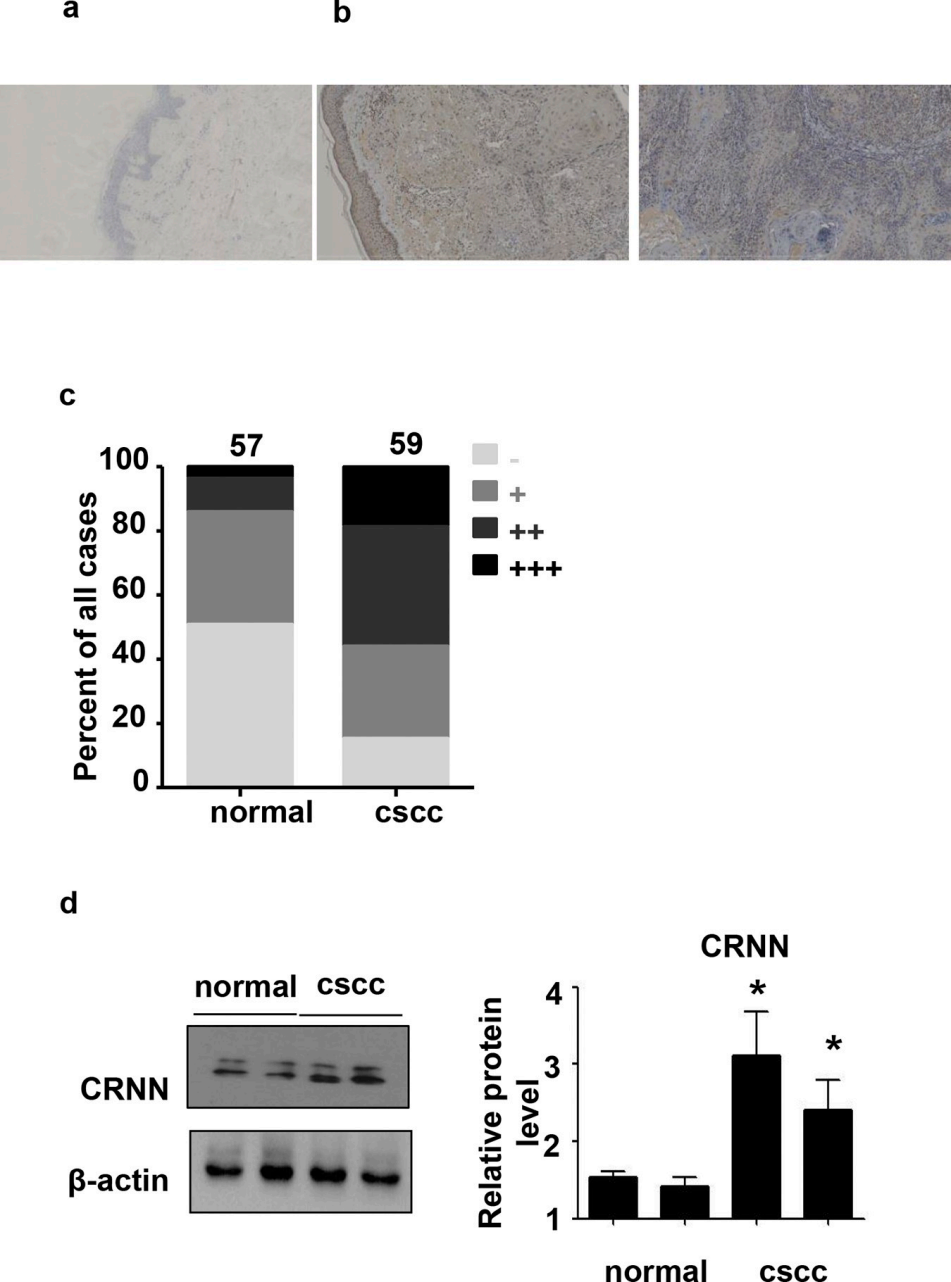
Distribution of CRNN in normal epithelium and lesions in cSCC patients. The researchers used formalin-fixed, paraffin-embedded specimens and performed staining with CRNN using Immunohistochemistry. (a) Normal skin (n = 57). Scale bar: 300 μm. (b) CSCC (n = 59). Scale bar: 300μm. (c) Semi-quantitative analysis of CRNN staining. (d) In the western blotting analysis of CRNN protein expression in normal skin samples and skin lesions of cSCC, the expression of CRNN protein is observed at 70 and 50 kDa. cSCC, cutaneous squamous cell carcinoma.

We initially examined the immunohistochemical expression of the CRNN protein in tissue samples obtained from patients with cSCC (Fig 1A and 1B). CRNN showed weak expression in the basal layer of 49.12% (n = 28/57) of normal skin tissue samples (Fig 1A) and strong expression in nearly all layers of cSCC samples, with a positive staining rate of 84.75% (n = 50/59, p< 0.005, Fig 1B). Overall, CRNN expression was higher in cSCC than in normal skin (Fig 1C). Furthermore, protein immunoblot analysis revealed that cSCC patients exhibited elevated levels of CRNN expression in their skin compared to the standard control group (Fig 1D).

### CRNN promotes keratinocyte growth via regulation of G1/S progression

**Fig 2:**
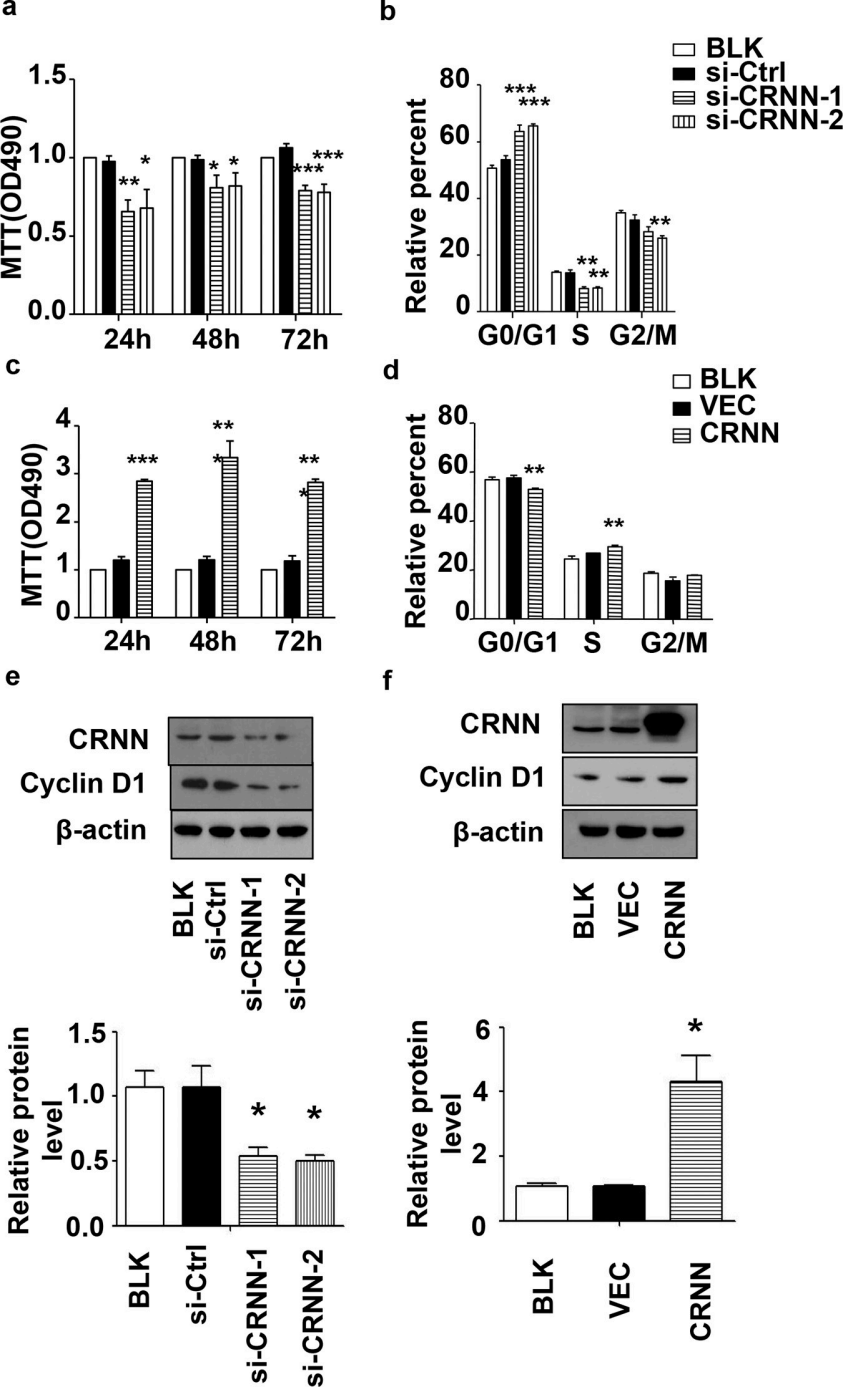
Effect of down-regulation and up-regulation of CRNN on cell proliferation. Cells were subjected to either CRNN siRNA transfection (a) or CRNN overexpression (c) and underwent MTT analysis after 24, 48, and 72 h. Flow cytometry analyzed the cell cycle after siRNA transfection (b) or CRNN overexpression (d) for 48 h. The expression of CRNN protein and cell cycle regulator cyclin D1. β-Actin as an internal control was analyzed by Western blotting analysis after CRNN knockdown(e) or overexpression (f), the expression of cyclin D1 protein is observed at 36 kDa. All quantitative data are presented as mean ± SEM (n = 3), *p < 0.05, **p < 0.01, ***p < 0.001.

Given the increased presence of CRNN in cSCC samples, it can be hypothesized that CRNN is involved in cSCC progression. We silenced CRNN with siRNA in SCL-1 cells to investigate its potential function. CRNN was knocked down using small interfering RNA (siRNA) in the SCL-1 cell line. Subsequently, MTT assay (Fig 2A) confirmed that CRNN depletion reduced cell growth in the SCL-1 cell line. For cell cycle analysis, propidium iodide (PI) was used to demonstrate the effect of CRNN knockdown on cell cycle arrest at the G1/S phase (Fig 2B). Concomitant with this effect, the expression of cyclin D1, a cell cycle regulator, decreased (Fig 2E). Collectively, our analysis of the impact of reduced CRNN functionally indicates that CRNN plays a crucial role in facilitating the transition from G1 to S phase in human cSCC.

To validate the findings of the loss-of-function analysis, we overexpressed CRNN in SCL-1 cells and analyzed its effect on the cell cycle. CRNN overexpression in SCL-1 cells enhanced cell proliferation, which was consistent with the results of the gene silencing study (Fig 2C). Overexpressing CRNN increased the number of cells in the S-phase (Fig 2D) and induced cyclin D1expression in the SCL-1 cell line (Fig 2F). Considering the combined findings from both loss-of-function and gain-of-function experiments, it can be inferred that CRNN plays a role in facilitating the transition from the G1 to S phase in cSCC cells.

### CRNN protects cSCC cells against apoptosis

**Fig 3:**
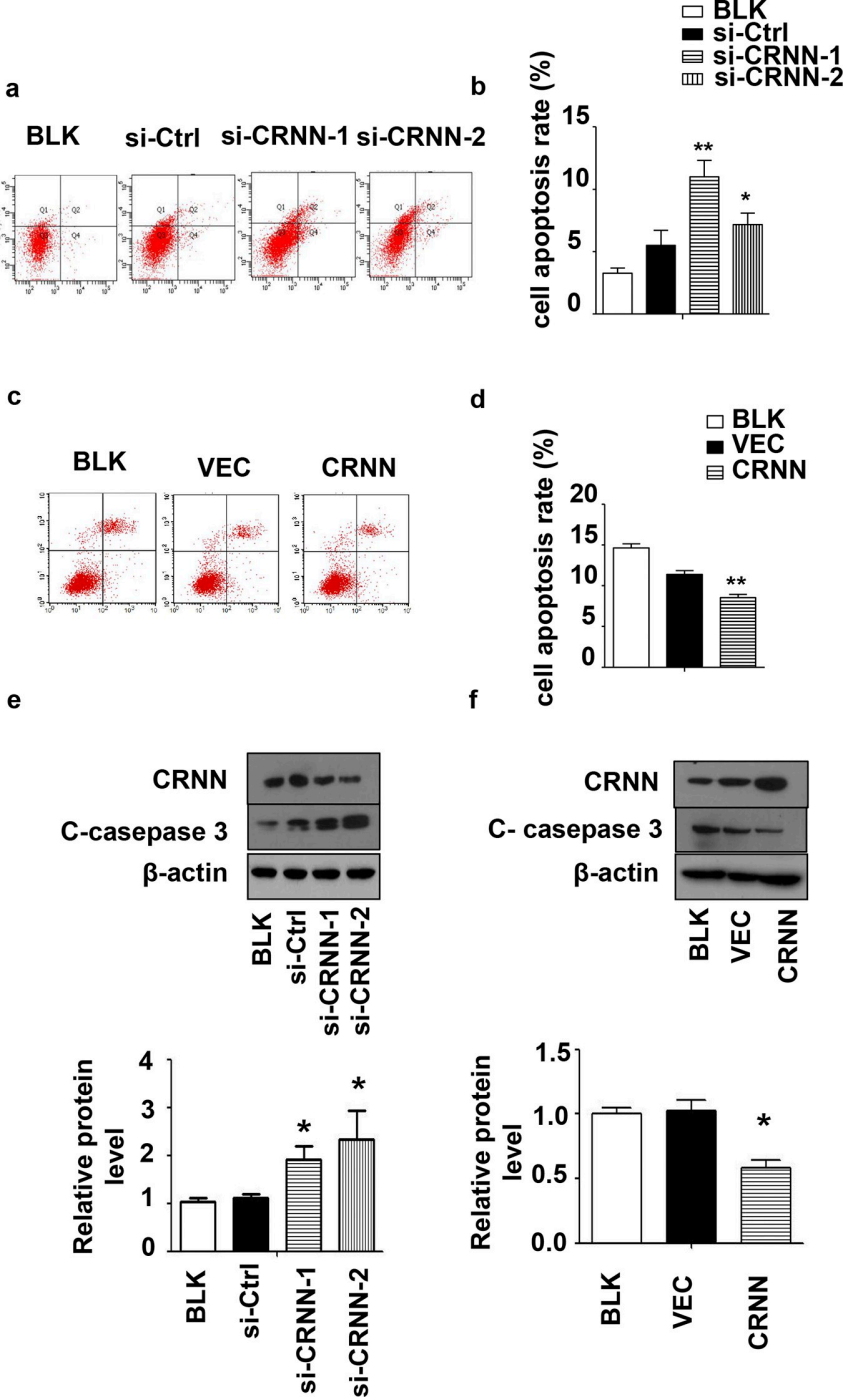
Effect of down-regulation and up-regulation of CRNN on cell apoptosis. (a) Cell apoptosis was assessed using Annexin V/PI staining and flow cytometry after introducing CRNN siRNA for 48 h. (b) Quantification of results from (a). (c) Cells were transfected with LV5-CRNN for 24 h, followed by treatment with 5 mg/mL 5-fluorouracil. The analysis of cell apoptosis was carried out 24 h after the treatment. (d) Quantification of results from (c). Cells were transfected with CRNN siRNA (e) or LV5-CRNN (f) for 48 h, and the Western blotting technique was employed to analyze the protein levels of cleaved caspase-3, the expression of C-caspase-3 protein is observed at 32 kDa.β-Actin was used as an internal control. All quantitative data are presented as mean±SEM (n = 3). *p < 0.05, **p< 0.01. BLK, blank transfected group; Ctrl, control; si, small interfering.

Annexin V/PI staining was conducted to assess cell apoptosis and determine the regulatory function of CRNN. In the control group, the initial apoptosis rate ranged from 3% to 6% in SCL-1 cells. However, CRNN knockdown resulted in an increased cell apoptosis rate, ranging from 8% to 13% (Fig 3A and 3B). Increased cell apoptosis was accompanied by more pronounced caspase-3 cleavage, indicating the activation of the apoptotic pathway (Fig 3E). To evaluate the effect of CRNN overexpression on cell death, apoptosis was induced using 5-fluorouracil (5-Fu) treatment, considering the relatively low initial level of apoptosis in the SCL-1 cell line. As expected, 5-Fu treatment induced apoptosis in the blank control (15.65%) and vector control (12.25%) groups. CRNN overexpression decreased the apoptosis rate of SCL-1 cells (7.71%) (Fig 3C and 3D) and caspase-3 cleavage (Fig 3F). In summary, CRNN acts as a protective factor for cSCC cells against apoptosis triggered by either basal cell damage or exposure to 5-Fu chemotherapy drug.

### CRNN promotes the migration/invasion ability of SCL-1 cells

**Fig 4:**
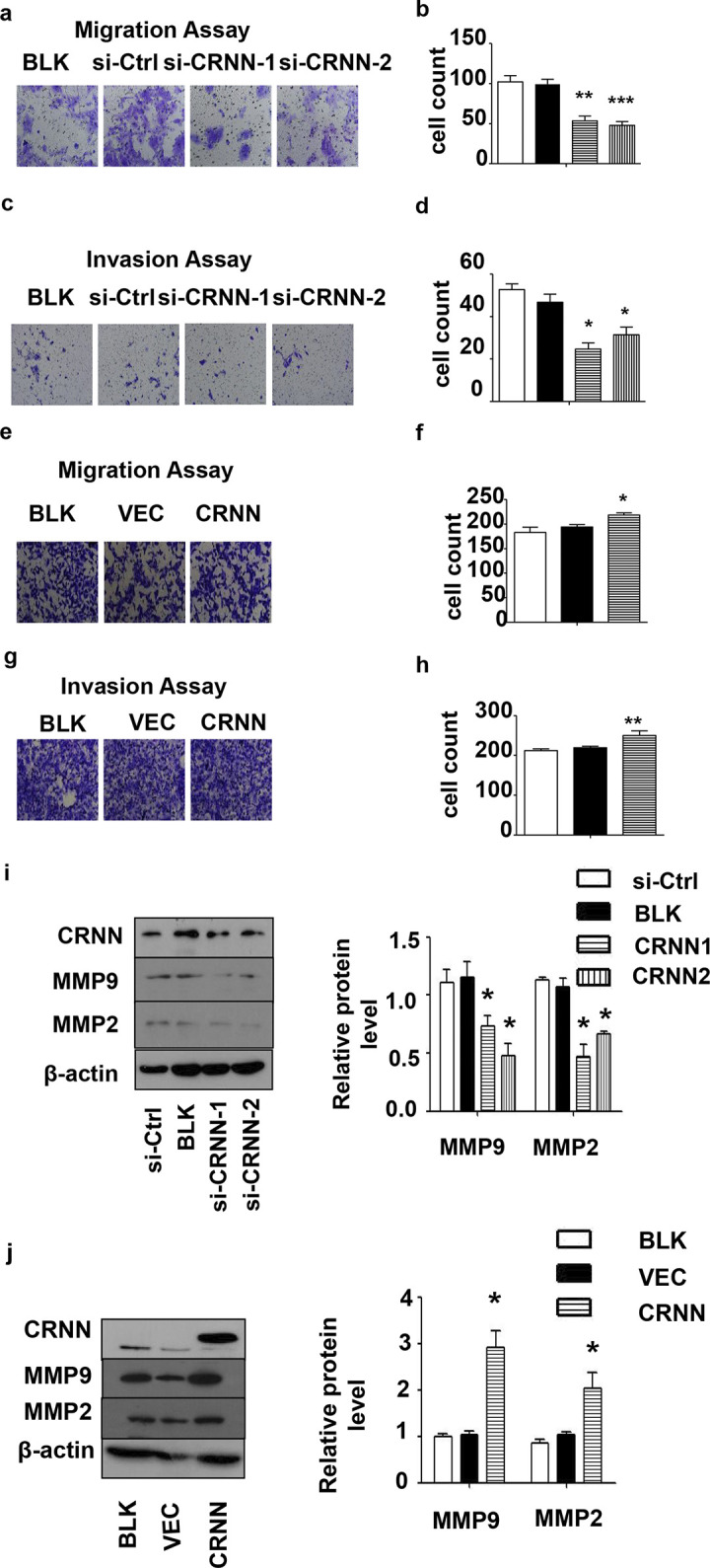
The effect of CRNN downregulation on migration and invasion ability in cSCC cells. Transwell assays were conducted to assess (a) cell migration or (c) invasion after transfection with CRNN siRNA for 48 h. Scale bar = 100μm. (b) Quantification of results from (a). (d) Quantification of results from (c). Transwell assays were performed to measure (e) cell migration or (g) invasion after transfection with LV5-CRNN for 48 h. Scale bar = 100 μm. (f) Quantification of results from e. (h) Quantification of results from g. (i) Western blotting analysis of MMP-2 and MMP-9 protein expressions after CRNN knockdown and LV5-CRNN transfection for 48 h, using β-Actin as an internal control. (j) Quantification of results from (i), the expression of MMP9 and MMP2 protein is observed at 78 and 72 kDa, respectively. All quantitative data are presented as mean±SEM (n = 5). *p < 0.05, **p < 0.01, ***p < 0.005. BLK, blank transfected group; MMP, matrix metallopeptidase; si, small interfering.

The Transwell assay (EMD Millipore, Billerica, MA, USA) was employed to examine the impact of CRNN on the in vitro migration and invasion of SCL-1 cells. Depletion of CRNN resulted in a notable decrease in the migratory and invasive capabilities of SCL-1 cells (Fig 4A–4D), whereas CRNN overexpression had the opposite effect (Fig 4E–4H). The role of the matrix metalloproteinase (MMP) family in tumor invasion and metastasis is widely recognized [27]. Therefore, we further detected the expression of MMP-2 and MMP-9, the two major MMPs involved in migration and invasion, respectively, in CRNN-depleted cells. The results depicted that CRNN downregulation decreased the expression of MMP-2 and MMP-9 (Fig 4I), whereas CRNN overexpression produced the opposite result (Fig 4J). The collected data correlated with the findings derived from the Transwell assay. Our results indicate that CRNN might enhance the migratory and invasive properties of cSCC cells by modulating of MMP-2 and MMP-9 expression.

### CRNN promotes keratinocyte growth by activating AKT signaling

**Fig 5:**
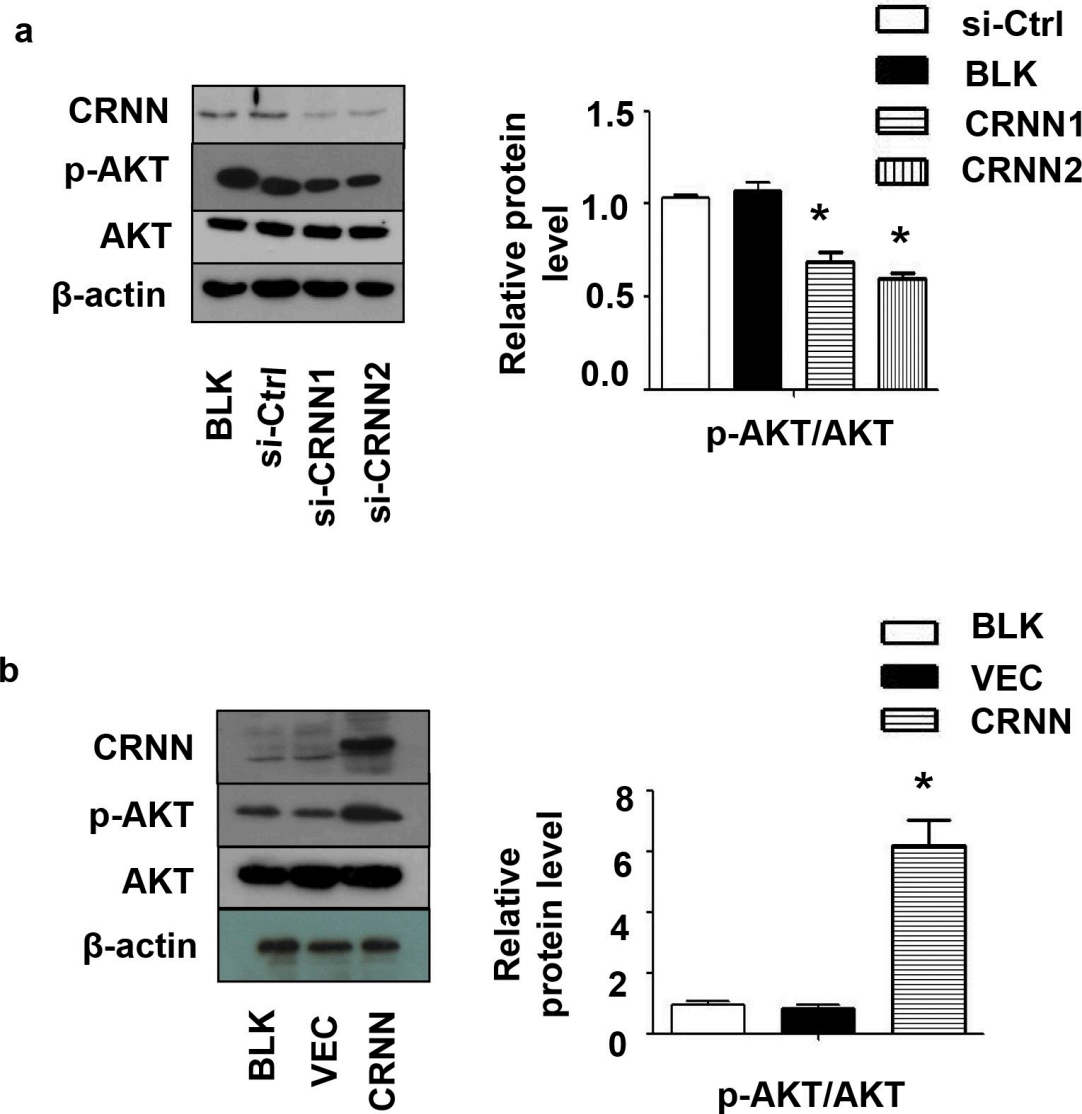
The effect of CRNN downregulation on AKT signaling pathway. Western blotting analysis was performed to detect the levels of CRNN, total AKT, and phospho-AKT in cells that were transfected with CRNN siRNA and LV5-CRNN, using β-Actin as an internal control, the expression of AKT and p-AKT protein is observed at 60 kDa.

To understand the role of CRNN in cSCC, we investigated the principal pathway responsible for regulating cell growth and survival: the PI3K/AKT pathway. PI3K catalyzes the production of a molecule known as PIP3, which in turn triggers the activation of Akt via PDK-1 [28]. PI3K is crucial for integrating various signals originating from growth factor receptors, innate immunity, and metabolites, ultimately coordinating the activation of Akt and other vital pathways [29]. CRNN Knockdown resulted in downregulation of these pathways, leading to a decrease in the levels of phospho-AKT and total AKT (Fig 5A). Conversely, CRNN overexpression increased the activity of the pathway, as evidenced by the increased levels of phospho-AKT (Fig 5B). The results of our study suggest that the CRNN gene contributes to the enhancement of keratinocyte proliferation by activating the AKT signaling pathway.

### CRNN upregulation promotes cSCC cell growth in vivo

To investigate the impact of CRNN on the growth of in vivo cSCC tumors, we implanted with LV-CRNN SCL-1 cells under the skin of the left leg and LV-VEC SCL-1 cells under the skin of the right leg, respectively, and monitored tumor development over time.

**Fig 6:**
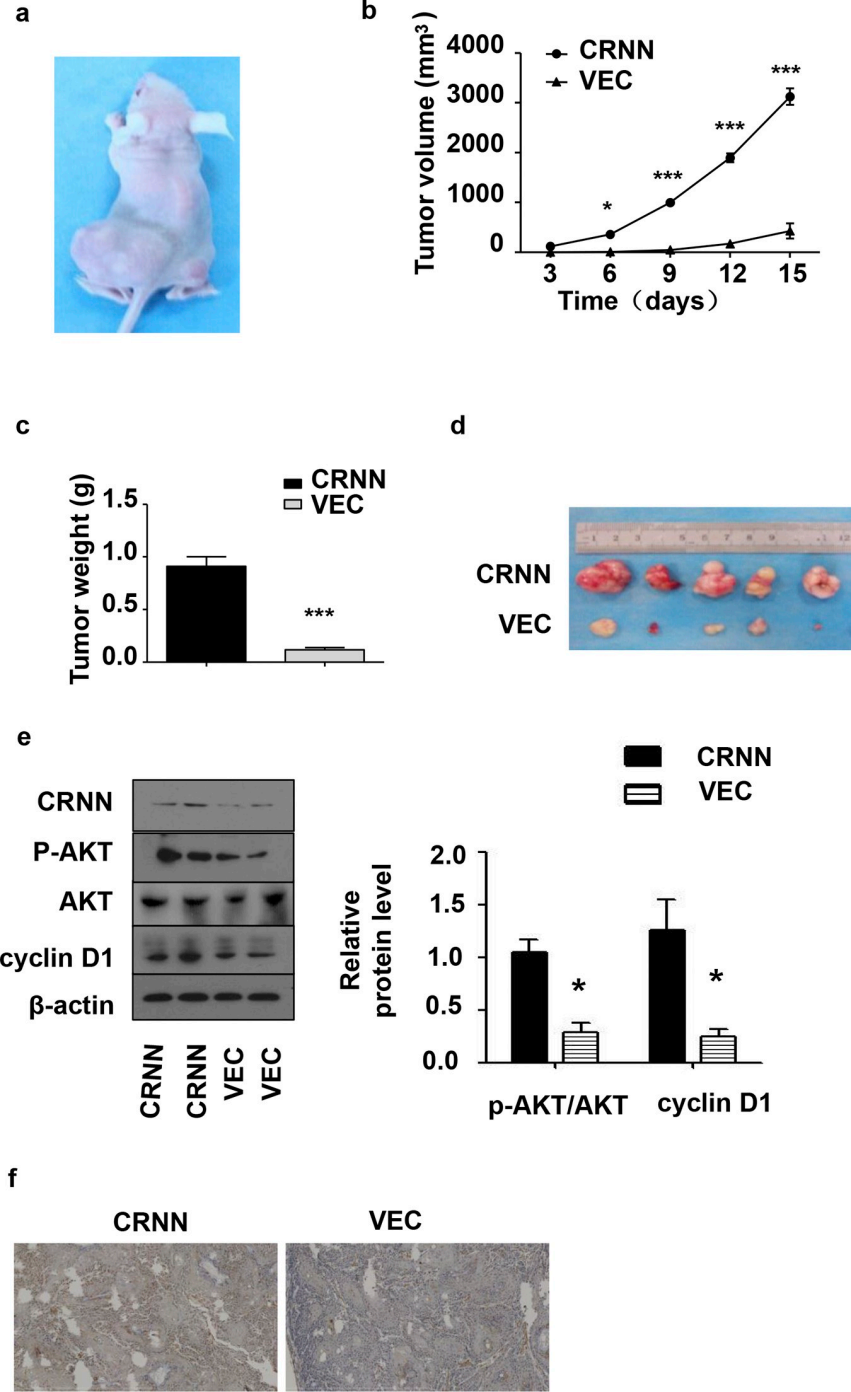
CRNN over-expression promotes the growth of cSCC xenografts in vivo. (a) Representative images of the treated mice are displayed with SCL-1 cells and transfected with LV5-CRNN (left) or LV5-VEC (right) for 15 days. Each group n = 5. (b) Growth curve of LV5-CRNN or LV5-VEC xenografts containing tumors in the nude mouse model. (c) Comparison of the average weight (n = 5) of LV5-CRNN or LV5-VEC xenografts at the endpoint. (d) Comparison of tumor size from LV5-CRNN or LV5-VEC xenografts at the end of the experiment (day 15). (e) Western blotting analysis of CRNN, phospho-AKT, AKT, and cyclin D1 expression in LV5-CRNN or LV5-VEC xenografts. β-Actin was used as an internal control, AKT and p-AKT protein expression is observed at 60 kDa, and Cyclin D1 protein is observed at 36 kDa. (f) Formalin-fixed xenografts were paraffin-embedded and stained with CRNN. Immunohistochemistry: LV5-CRNN (left) and LV5-VEC (right). Scale bar: 300 μm. The data are presented as mean ± SEM (n = 5). *p <0.05, ***p <0.001.

In line with the findings observed in the laboratory setting, in vivo overexpression of CRNN significantly promoted tumor growth (Fig 6A). At the end of the 15-day study, tumors from the LV-CRNN group exhibited considerably larger dimensions and greater mass than those from the control group (Fig 6B–6D). Western blotting confirmed the superior efficiency of the LV-CRNN in achieving CRNN overexpression at the endpoint. As expected, the phospho-Akt level in the tumor tissues of the LV-CRNN group was increased (Fig 6E). Additionally, we examined the presence of CRNN protein in tissue samples from nude mouse tumor tissues using immunohistochemical staining (Fig 6F).

## Discussion

The CRNN gene, which is involved in the differentiation of skin cells, is located on chromosome 1q21, where the epidermal differentiation complex is also situated [10, 11]. Recent studies have demonstrated that CRNN are present in various human squamous cell carcinomas. Previous research has demonstrated that the PI3K-Akt pathway can enhance the proliferation of keratinocytes in psoriasis, with CRNN playing a facilitating role [30]. However, the precise involvement of this factor in cSCC progression remains unclear. Our research suggests that the expression of CRNN is closely linked to the progression of cSCC and that CRNN triggers the activation of the AKT signaling, thus enhancing the proliferation and survival of epidermal cells. Furthermore, we observed increased expression of CRNN in skin lesions of patients diagnosed with cSCC.

In cutaneous SCC, Jia et al. reported a significant reduction in YAP expression compared to normal skin tissue. Their study revealed that YAP controls the progression of cSCC cells by governing the G1/S transition [31]. Imai et al. demonstrated that CRNN upregulation led to the arrest of the G1-phase cell cycle and suppression of cyclin D1 in human oral squamous cell carcinoma [12]. Furthermore, inhibition of G1/S checkpoint cell cycle arrest in esophageal squamous cell carcinoma is achieved by upregulating P21WAF1/CIP1 and Rb expression by CRNN [25]. Through regulation of the G1/S transition, our study provides evidence showcasing the active role of CRNN in facilitating the proliferation of cSCC. CRNN knockdown decreased the expression of various factors that regulate the transition from the G1 to S phase, including cyclin D1.

Moreover, suppressing CRNN hindered Akt activation. However, Karumuri et al. observed a significant five-fold decrease in the expression of the studied factor in histologically advanced tumors compared to histologically well-differentiated tumors [32]. Karumuri et al. found a negative correlation between the expression levels of CRNN protein and lymph node metastasis [33]. This finding contradicts our observed studies and may be attributed to potential issues with our sample size. Thorough research is necessary due to the controversial nature of this matter.

Despite considerable progress in understanding the mechanisms underlying cSCC development in recent years, Xiao et al suggested that TXNDC9 plays an important biological role in cSCC progression [34], and there is a lack of viable treatment options for this condition. Tellado et al treated 52 cSCC patients of the nasal plane using Electrochemotherapy. The recovery of appetite after treatment was significantly reduced, and the local response and overall survival rates were similar to those in the control group [35]. Moreover, the suppressing of CRNN expression decelerates the proliferation of skin cells. Our findings strongly support the crucial role of CRNN in cSCC and suggest a promising therapeutic target with wide-ranging clinical implications, underscoring the significance of our study. Low-differentiated cSCC is rare, and no healthy keratinocytes experiments were performed in our study, to understand the mechanism of CRNN in the occurrence and development of skin squamous cell carcinoma.

## Supporting information

S1 Raw images (PDF)
