# Supplementary figures and images for: The role of cornulin (CRNN) in the progression of cutaneous squamous cell carcinoma involving AKT activation in SCL-1
Source: PLoS One. 2024 Sep 18;19(9):e0308243. doi: 10.1371/journal.pone.0308243 (PMC11410236; doi:10.1371/journal.pone.0308243)

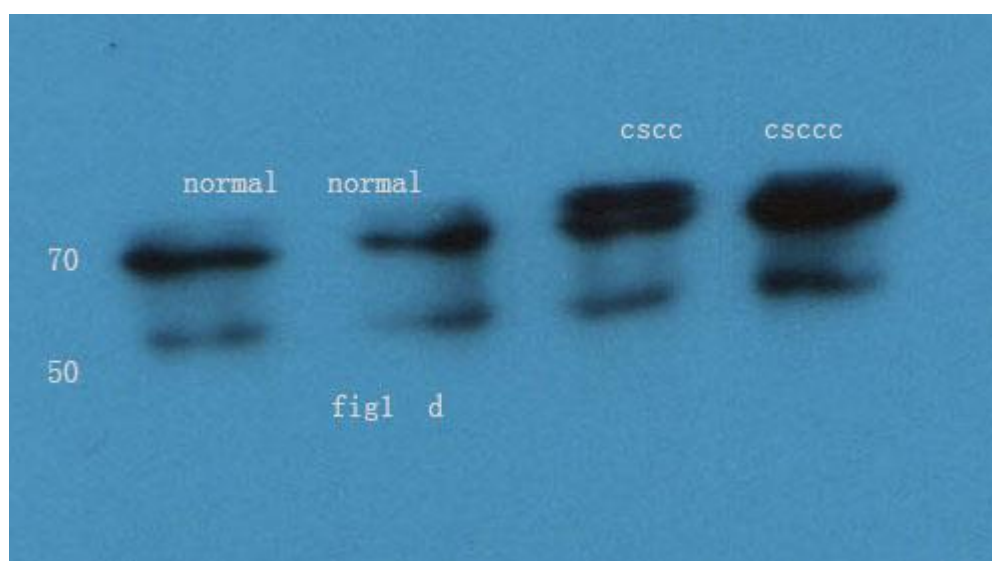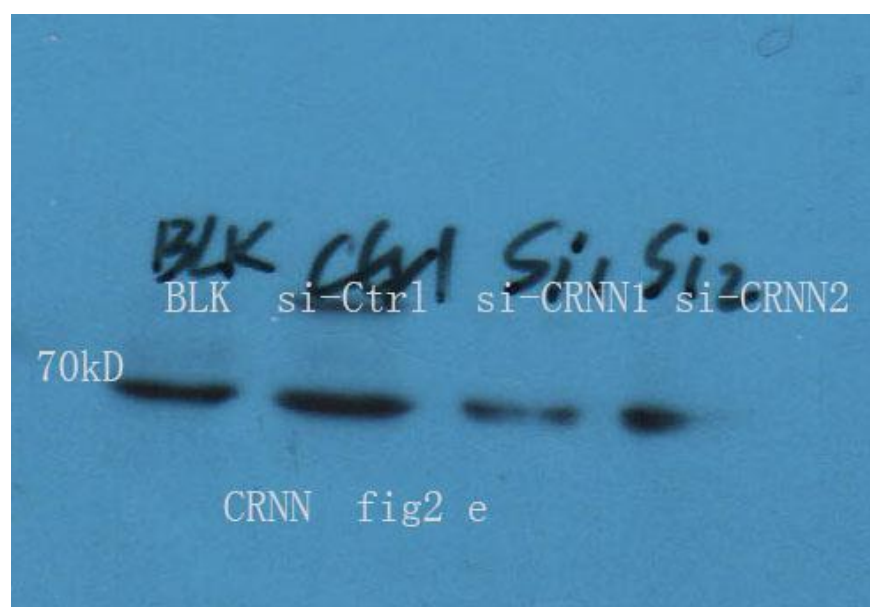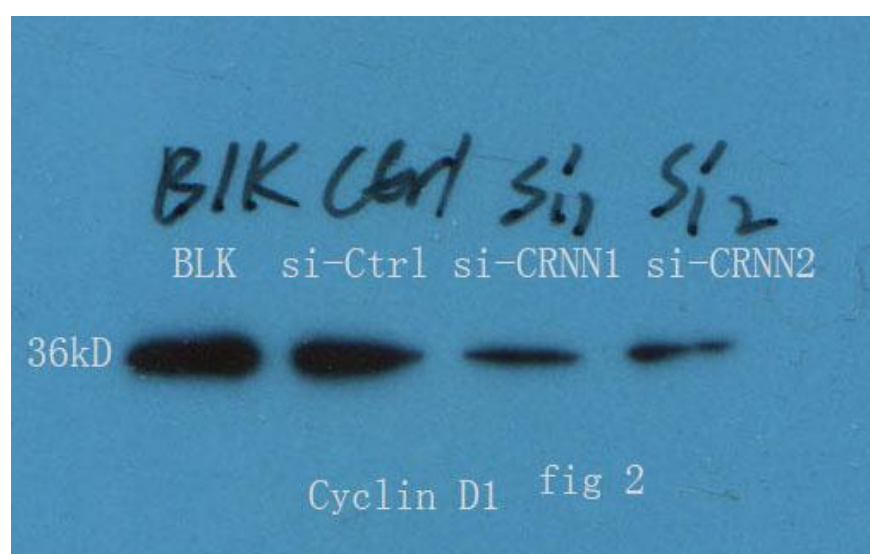

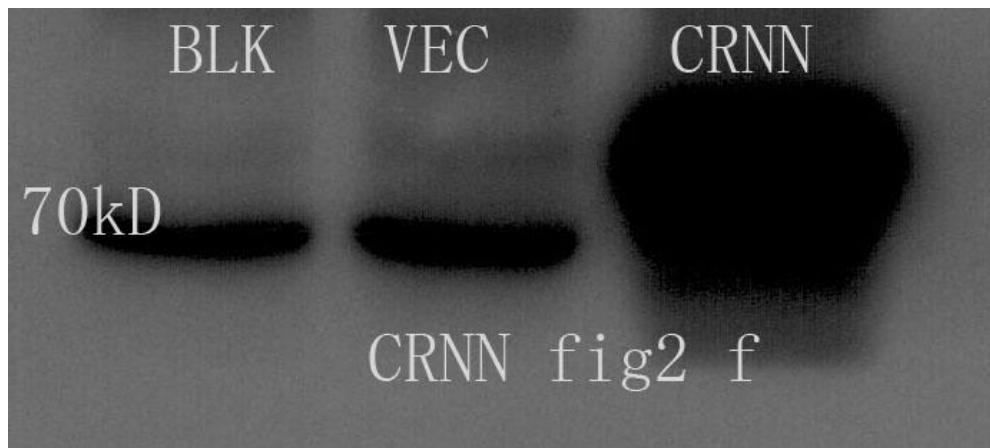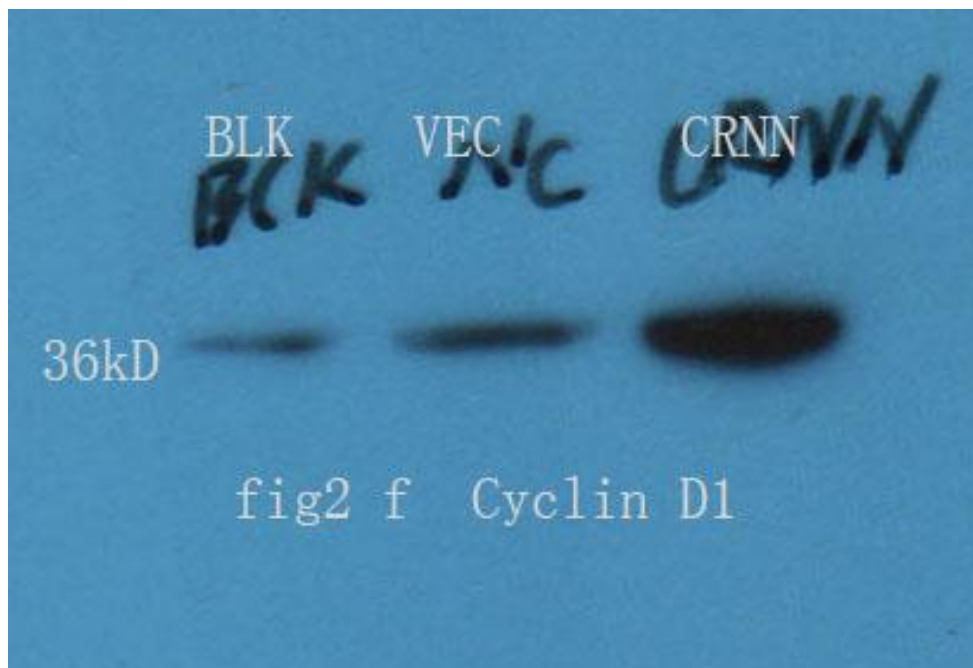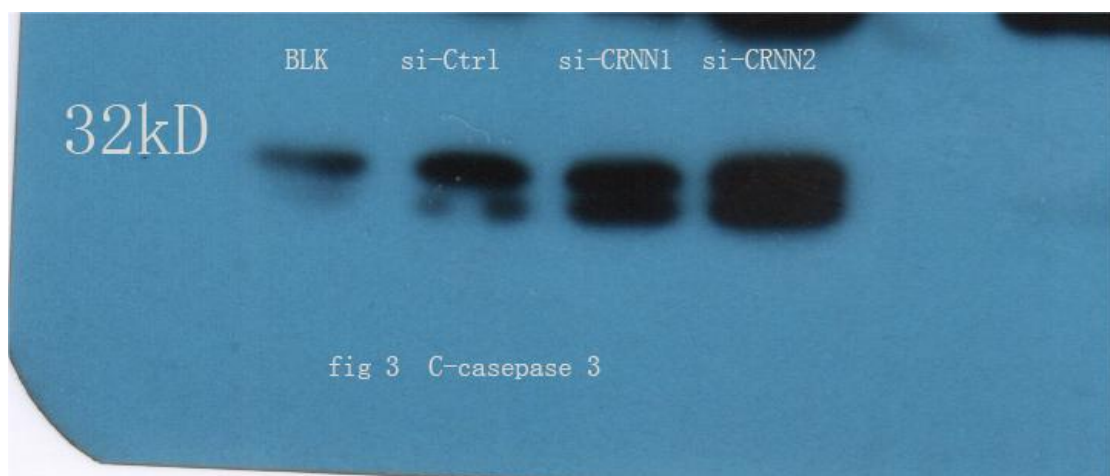

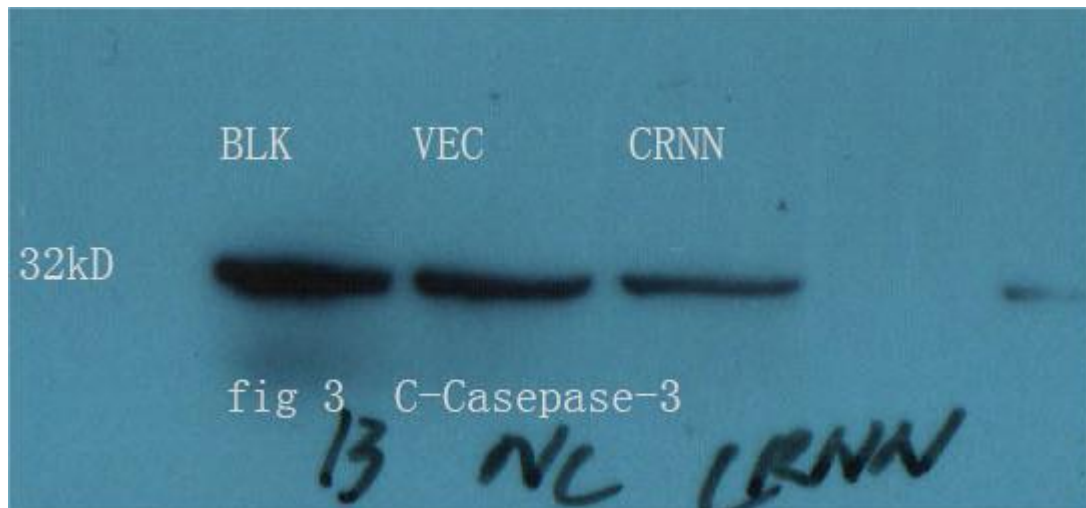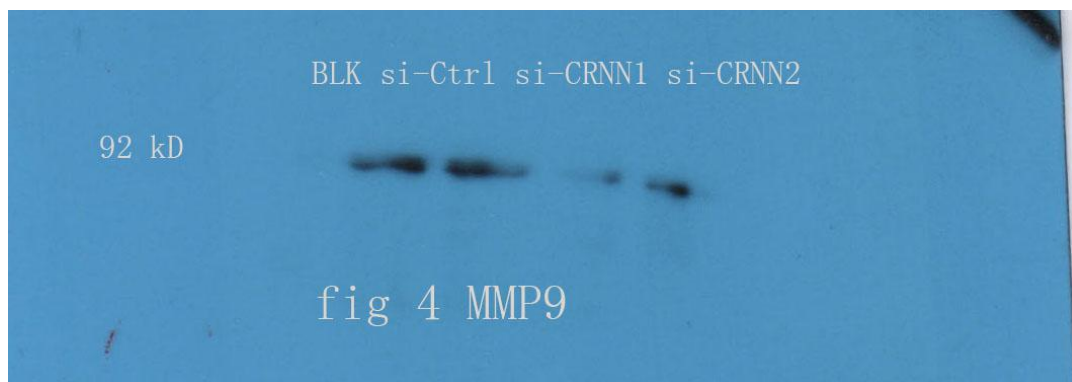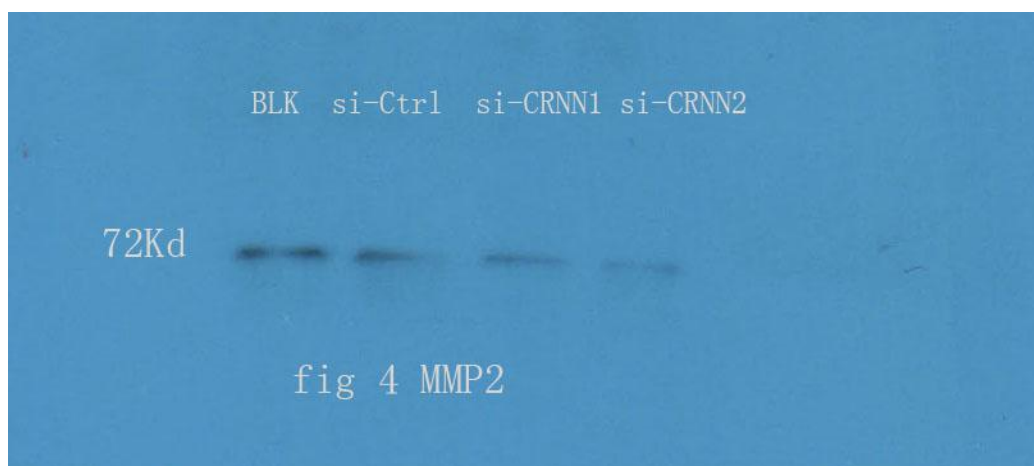

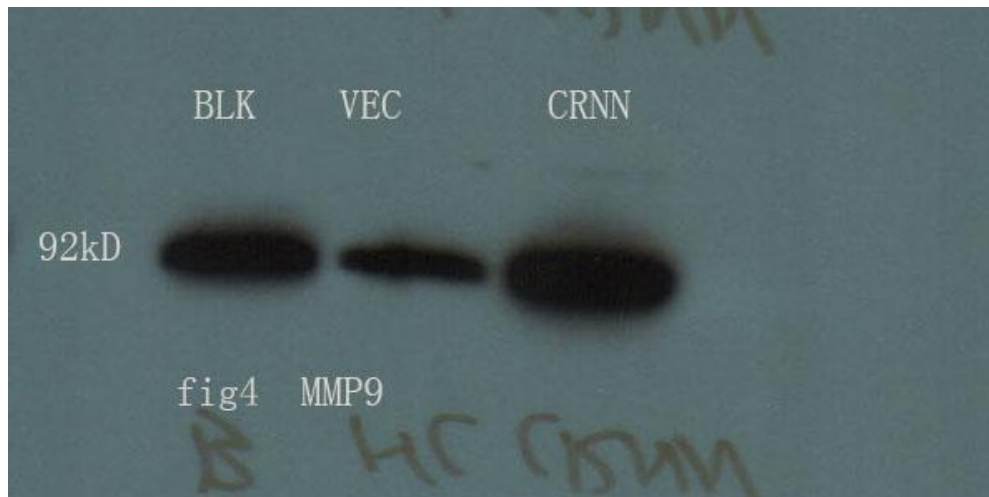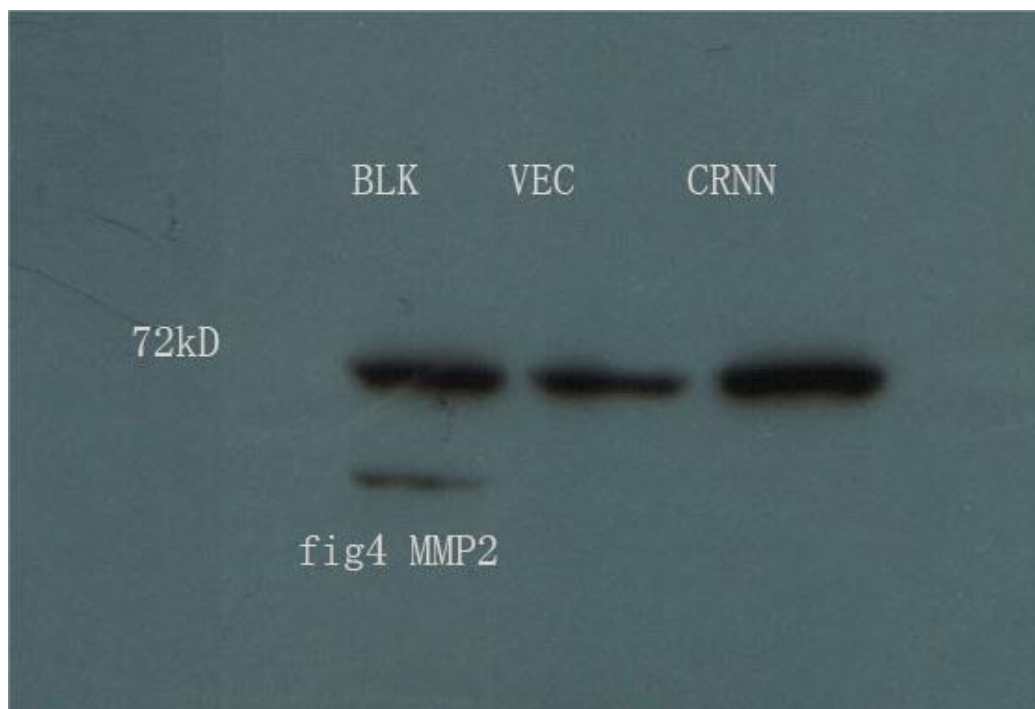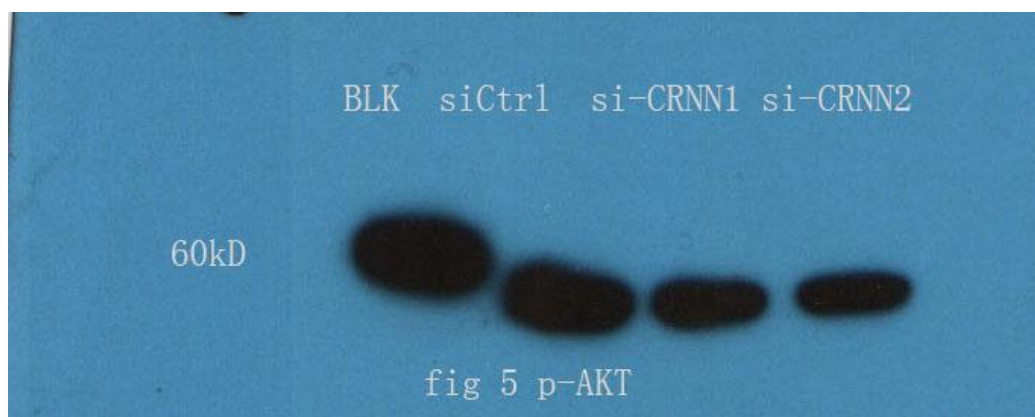

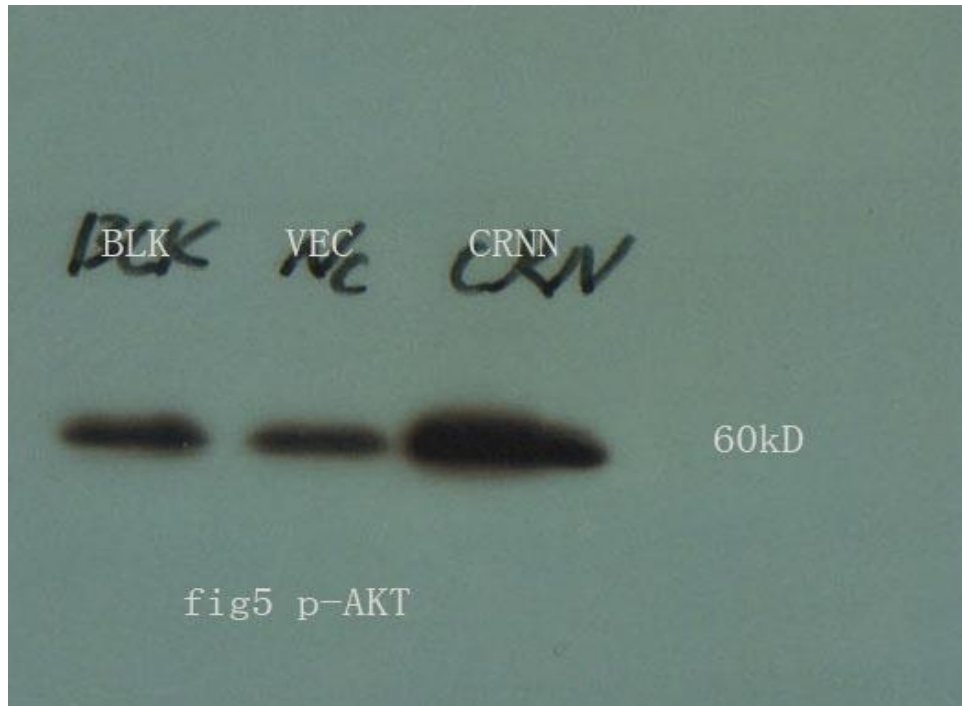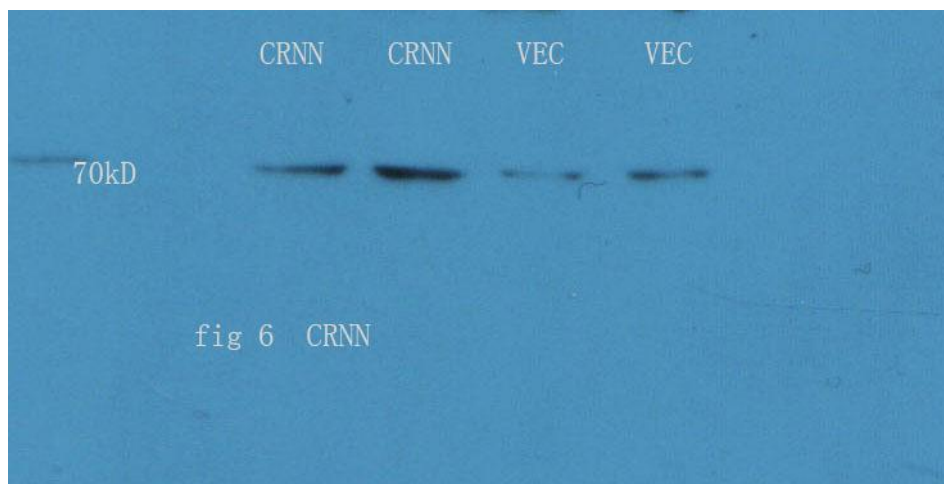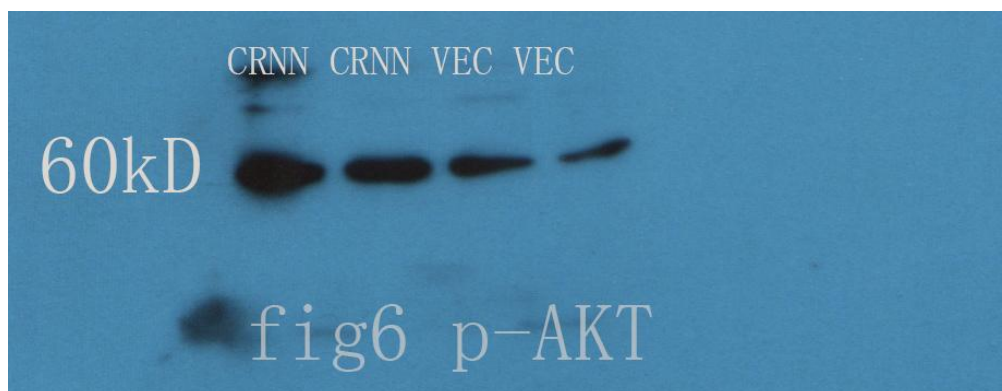

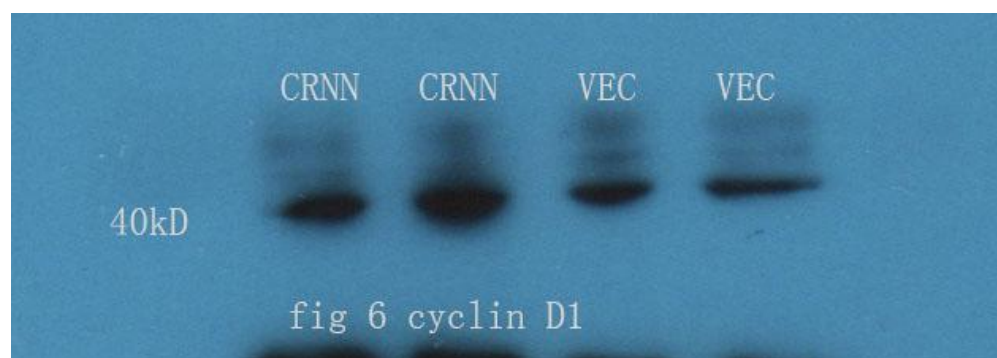

Supplement: S1 Raw images — (PDF) [file pone.0308243.s001.pdf]
